# A Meta-Analysis of Multiple Whole Blood Gene Expression Data Unveils a Diagnostic Host-Response Transcript Signature for Respiratory Syncytial Virus

**DOI:** 10.3390/ijms21051831

**Published:** 2020-03-06

**Authors:** Ruth Barral-Arca, Alberto Gómez-Carballa, Miriam Cebey-López, Xabier Bello, Federico Martinón-Torres, Antonio Salas

**Affiliations:** 1 Unidade de Xenética, Instituto de Ciencias Forenses, Facultade de Medicina, Universidade de Santiago de Compostela, and GenPoB Research Group, Instituto de Investigaciones Sanitarias (IDIS), Hospital Clínico Universitario de Santiago (SERGAS), 15706 Galicia, Spain; barralarcaruth@gmail.com (R.B.-A.); ksl8tr@gmail.com (A.G.-C.); cebeymiriam@hotmail.com (M.C.-L.); xbello@gmail.com (X.B.);; 2 Translational Pediatrics and Infectious Diseases, Department of Pediatrics, Hospital Clínico Universitario de Santiago de Compostela, 15706 Galicia, Spain; 3 Genetics, Vaccines and Infections Research Group (GENVIP), Instituto de Investigación Sanitaria de Santiago, 15706 Santiago de Compostela, Spain

**Keywords:** meta-analysis, RNA, transcriptomic, RSV, respiratory syncytial virus, array

## Abstract

Respiratory syncytial virus (RSV) is one of the major causes of acute lower respiratory tract infection worldwide. The absence of a commercial vaccine and the limited success of current therapeutic strategies against RSV make further research necessary. We used a multi-cohort analysis approach to investigate host transcriptomic biomarkers and shed further light on the molecular mechanism underlying RSV-host interactions. We meta-analyzed seven transcriptome microarray studies from the public Gene Expression Omnibus (GEO) repository containing a total of 922 samples, including RSV, healthy controls, coronaviruses, enteroviruses, influenzas, rhinoviruses, and coinfections, from both adult and pediatric patients. We identified > 1500 genes differentially expressed when comparing the transcriptomes of RSV-infected patients against healthy controls. Functional enrichment analysis showed several pathways significantly altered, including immunologic response mediated by RSV infection, pattern recognition receptors, cell cycle, and olfactory signaling. In addition, we identified a minimal 17-transcript host signature specific for RSV infection by comparing transcriptomic profiles against other respiratory viruses. These multi-genic signatures might help to investigate future drug targets against RSV infection.

## 1. Introduction

Respiratory syncytial virus (RSV) is the main cause of lower respiratory tract infections in early life [1], and one of the major causes of morbidity and mortality, especially in children younger than six months [2]. Re-infections are also common in patients older than two years and adults, although usually less severe [3,4]. Patients with premature birth, chronic lung disease, congenital heart disease, and immunodeficiency are more likely to suffer from severe forms of RSV and may require hospitalization [5]; however, most of the children hospitalized with severe RSV disease lack known identifiable risk factors [6,7].

It has been shown that host genetic susceptibility to the disease may play a key role in the different pathogenesis produced by RSV in children [8,9], but many aspects of the host-pathogen interaction remain unknown [10]. The notable impact of pediatric respiratory diseases produced by RSV in the health system raises the need for a more in-depth understanding of the molecular mechanism responsible for the host susceptibility to the infection. This would improve the development of new preventive, diagnostic, and therapeutic strategies, which could reduce the social, medical, and economic burden of the disease [11].

In the era of personalized medicine and molecular diagnostics, the study of host transcriptomics during infection is becoming an important tool not only for the study of the host-pathogen interaction but also for the diagnosis and prognosis of diseases. In the last few years, some host expression signatures related to both infectious [12,13,14,15,16] and non-infectious diseases [17,18] have been reported. However, most of the studies are focused only on particular cohorts from a certain ethnicity or population group and restricted geographic areas, from a specific age group and the same epidemic period. Moreover, these studies compare patients against healthy controls without investigating the impact of other respiratory pathogens on the host transcriptome.

Moreover, the technical noise inherent to microarray data, together with the usually small (underpowered) cohorts analyzed in individual studies, often lead to unreliable, inconsistent and non-reproducible results, probably due to type I and type II errors [19,20]. Instead, a multi-cohort study allows increasing sample size and statistical power, leading to more accurate and conclusive findings and, thus, facilitating the implementation of the results into a molecular test [21,22,23,24].

By compiling different datasets from respiratory diseases-related host transcriptomic studies available in public resources we aimed at investigating specific expression signatures allowing to differentiate RSV respiratory infection from other viral respiratory infections and also healthy controls. We also explored the role of the differentially expressed genes (DEG) in RSV-infected patients and related pathways in the context of RSV infection.

## 2. Results

### 2.1. Differentially Expressed Transcripts between RSV and Healthy Controls

A principal component analysis (PCA ) of the combined data (RSV samples and healthy controls) was carried out to detect batch effects in the eight datasets analyzed. This analysis revealed that the transcriptomes of the RSV cases in the GSE80179 form a separate cluster when compared to the rest of the samples (Figure S1). This anomalous pattern could be explained by different severities, strand or sampling time points with regard to the other samples, or undesirable batch effects. This dataset was therefore eliminated from further analysis.

Thus, the meta-analysis was finally conducted on a total of seven datasets containing transcriptomic data from 922 samples: 296 RSV, 4 coronaviruses, 4 enteroviruses, 188 influenzas, 71 rhinoviruses, 93 coinfections, and 266 healthy controls; from both adult and pediatric patients (Table S1).

**Figure 1:**
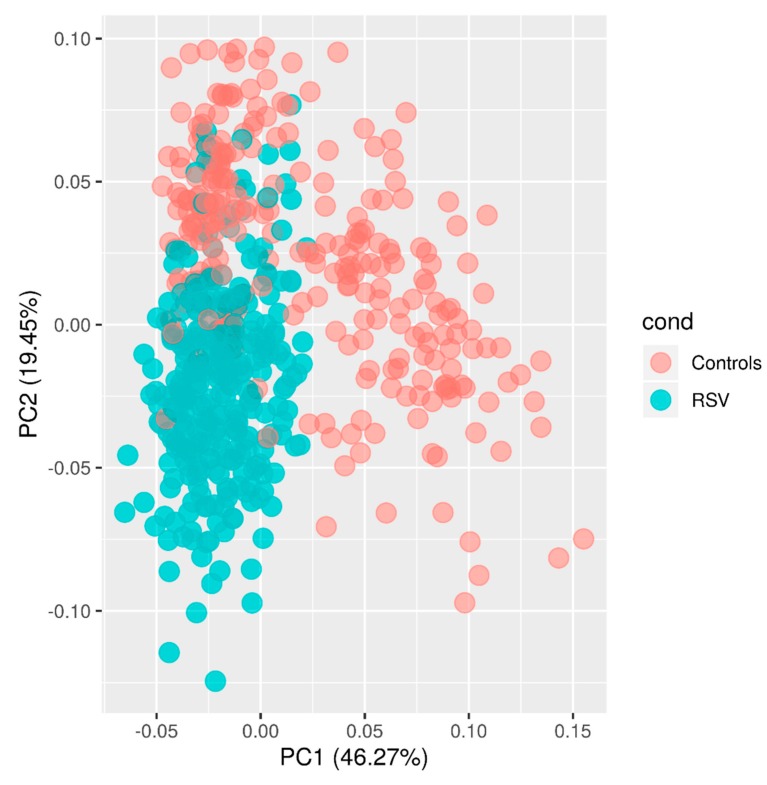
PCA of transcriptome profiles for the seven datasets used for the meta-analysis. Two first principal components (PC1 and PC2) are shown.

A new PCA was conducted with the remaining seven datasets and based on the 100 most DEG (Figure 1). The first principal component (PC1; accounting for ~46% of the variation), in combination with PC2 (accounting for ~19% of the variation), separates all the samples in two clusters according to their disease status. It is remarkable that, while the healthy controls appear more scattered in the plot, RSV patients are more tightly clustered, suggesting that the RSV alters the transcriptome in a more specific manner. It is also noteworthy that the 93 cases of co-infection included in the meta-analysis do not generate a pattern of sub-clustering in the PCA plot.

By comparing the transcriptomes of RSV-infected patients to healthy controls, we identified 1,562 genes differentially expressed (678 over-expressed and 884 under-expressed; FDR of 5%; Table S2). This differential gene expression pattern explains the clustering pattern observed in the PCA (Figure 1).

### 2.2. Functional Enrichment Analysis

Our functional enrichment study using Reactome showed that the DEG were enriched in the host cell-cycle pathway in RSV infected patients when compared to controls (R-HSA-69620, adjusted *p*-value = 1.16 × 10^−5^; R-HSA-69278, adjusted *p*-value = 3.83 × 10^−5^; R-HSA-1640170, adjusted *p*-value = 2.37 × 10^−5^) (Table S3).

Among the DEG between RSV-infected patients and healthy controls, a number of them affecting immunologic-related pathways are strongly over-represented: (i) immune system (R-HSA-168256; adjusted p-value = 4.06 × 10^−27^), (ii) cytokine signaling in immune system (R-HSA-1280215: *STAT1*, *STAT2*, *MMP-9*; adjusted p-value = 7.90 × 10^−16^), (iii) innate immune system (R-HSA-168249; adjusted p-value = 5.92 × 10^−19^) and (iv) adaptive immune system pathways (R-HSA-1280218; adjusted p-value = 1.11 × 10^−7^). We also observed an over-representation of DEG belonging to pathways related to (i) interleukins signaling (R-HSA-449147; adjusted p-value = 1.36 × 10^−7^) such as interleukin-1 family signaling (R-HSA-446652; adjusted p-value = 1.45 × 10^−6^) and (ii) interleukin-1 signaling (R-HSA-9020702; adjusted p-value = 7.67 × 10^−6^; Table S3). RSV infection also causes an over-representation DEG related to the interferon signaling pathway (R-HSA-913531; Table S3) and interferon-inducible genes such as *EIF2AK2*, *MX1* and *IFITM1*. Over-representation is also observed in essential proteins of the innate immune response such as oligoadenylate synthase (OAS) family proteins and RNAse L, which are responsible for RNA degradation hereby blocking viral replication [25].

In addition, other pathways related to host response to infectious disease (R-HSA-5663205; adjusted *p*-value = 2.08 × 10^−21^) and others involved in the viral infection process, such as R-HSA-168254 (influenza infection; adjusted *p*-value = 2.56 × 10^−17^), R-HSA-162906 (HIV infection; adjusted *p*-value = 8.62 × 10^−7^), R-HSA-168273 (Influenza Viral RNA Transcription and Replication; adjusted *p*-value = 3.91 × 10^−17^) and R-HSA-192823 (Viral mRNA Translation; adjusted *p*-value = 5.14 × 10^−18^), were found to be clearly enriched in RSV patients (Table S3). RSV infection also provokes an over-representation of DEG related to the neutrophil degranulation pathway (R-HSA-6798695; adjusted *p*-value = 1.59 × 10^−14^).

RSV additionally induced over-representation of differential expressed pattern recognition receptor genes as compared to healthy controls (Table S3): (i) Toll-like receptors (TLR) cascades (R-HSA-168898; adjusted *p*-value = 1.23 × 10^−3^), including *TLR3* (R-HSA-168164; adjusted *p*-value = 2.45 × 10^−2^), which are specialized in the recognition of conserved molecular features of different pathogens such as bacteria, viruses, fungi, and parasites; and (ii) C-type lectin receptors (R-HSA-5621481; adjusted *p*-value = 5.12 × 10^−6^), capable of sensing glycans present in viral pathogens to activate antiviral immune responses such as phagocytosis, cytokine production, antigen processing and presentation, and subsequent T cell activation.

Related to this, AIM2-like receptors (interferon inducible protein AIM2; adjusted *p*-value = 7.90 × 10^−24^; Table S2) were found to be over-expressed (Table S2). AIM2-like receptors can act as enzymatic complexes (inflammasomes) and are known to be activated by the presence of microbial DNA within the cytosol [26]. In response to viral and bacterial infection these inflammasomes trigger caspase-1 activation and subsequently induce the release of mature pro-inflammatory interleukins such as IL-18, contributing to the immune response to pathogens [27] (Table S2).

Other enriched pathways (Table S3) are the *TLR4* cascade (R-HSA-166016), which acts as co-receptor with *CD14* (adjusted *p*-value = 2.29 × 10^−8^) detecting bacterial lipopolysaccharide, and other *TLR4*-related pathways such as *TRIF*(*TICAM1*)-mediated *TLR4* signaling (R-HSA-937061) and MyD88-independent *TLR4* cascade (R-HSA-166166).

Finally, we found that the RSV infection causes an under-representation of DEG related to the olfactory signaling pathway (R-HSA-381753; adjusted *p*-value = 1.09 × 10^−12^) (Table S3).

### 2.3. RSV-Specific Expression Signature

**Figure 2:**
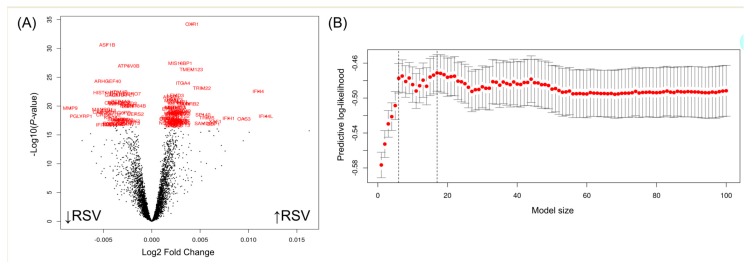
(**A**) Volcano plot of the genes differentially expressed when comparing respiratory syncytial virus (RSV)-infected children to children infected by other pathogens. The 100 most significant genes are highlighted, and these were used for the signature search. The upward arrow close to the x-axis indicates upregulated genes (log_2_ FC < 0), whereas the downward arrow indicates down-regulated genes (log_2_ FC > 0). (**B**) Optimal gene model size according to PReMS algorithm. The X axis represents the training set predictive log-likelihood, while the Y axis represents the number of genes. Solid grey bars indicate one standard error of the predictive log-likelihood. Vertical dashed lines show the optimal and one standard error fits as described in PreMS documentation. Red dots represent DEG that passed the significance threshold after a multiple test correction.

To investigate a specific RSV transcriptome signature, we compared RSV expression patterns against a viral multi-cohort set following a cross-validation strategy that randomly divides the whole dataset into a training and a test set. Representative samples of coronaviruses, influenzas, rhinoviruses, enteroviruses, and RSV transcriptomes were present in both the training and the test sets. The volcano plot of Figure 2A shows the genes differentially expressed when comparing RSV-infected children to children infected by other pathogens.

**Figure 3:**
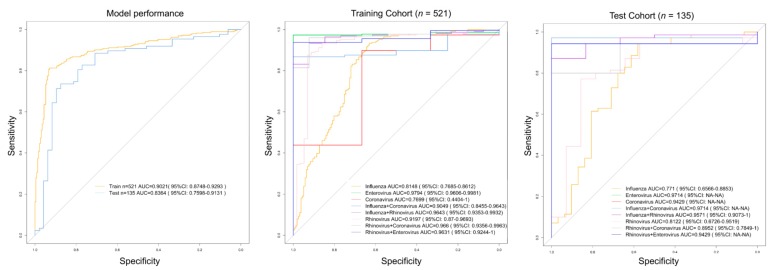
Receiver operating characteristic curves (ROC) based on the specific RVS 17-transcript signature. (**A**) ROC curve for the training and test sets (all pathogens vs. RSV). (**B**) ROC curves for each pathogen in the training cohort. (**C**) ROC curves for each pathogen in the test cohort.

Among the 100 most significant genes in this analysis (indicated in red in Figure 2A), we searched for the minimum transcriptome signature allowing to discriminate between RSV from other pathogens using the optimal gene model size according to PReMS algorithm. Figure 2B shows the optimal model characterized by 17 genes that conform to an optimal signature to distinguish RSV from other viral conditions. This 17-transcript signature (Table 1; Figure 3A) identified in the discovery training set distinguished RSV from other viral conditions with area under de curve (AUC) of 90.2% (95%CI: 87–93.0), sensitivity of 81.3% and specificity of 93.0%.

When applying this signature to the test set, the AUC decreases to 83.6% (95%CI: 76.0–91.3), with a sensitivity of 73.6% and specificity of 87.5%.

When we evaluated the performance of the model to differentiate RSV from each pathogen individually, we obtained AUC values ranging from 77.0% for coronavirus, to 97.9% for enterovirus in the training cohort, and values ranging from 77.1% for influenza to 97.1% for enterovirus in the test cohort (Table 2; Figure 3B,C).

## 3. Discussion

In spite of the global burden of RSV, our knowledge of how RSV interacts with the host remains incomplete. Moreover, the study of the host gene expression response to RSV infection is usually restricted to specific and very homogeneous cohorts, hampering the interpretation of the results, the impact of the conclusions obtained and their translation into a molecular test that could be used in routine clinical practice. The present meta-analysis shows that RSV infection leads to global changes in the host transcriptome, affecting not only the transcriptomic machinery of the airway infected cells [28], but also the expression of hundreds of genes in blood cells (Table S2). In particular, according to our results, RSV alters the expression of >1,500 genes in the host compared to healthy controls, involving e.g., cell cycle and immune system genes. This host response can be differentiated to those from other viruses with only 17-transcripts, which might eventually facilitate its use for clinical diagnosis.

In mammals, the cell cycle is governed by a complex molecular machinery. The data indicate that patients infected with RSV have an over-representation of differentially expressed cell cycle-related genes, a well-known strategy employed by many other viruses to facilitate their replication [29,30].

As expected, we observed DEG and pathways related to the immunological response that are particularly relevant in the context of RSV infection, e.g., cytokines, which are small proteins released by cells that are known to play a major role in interactions and communications between cells. RSV infection causes a significant alteration of cytokine pathways and related pathways (adjusted *p*-value = 7.90 × 10^−16^; Table S3), a finding that is in agreement with other authors, indicating that host genetic variation in cytokines predisposes to suffering complications during RSV infection [31]. Although more studies are needed to confirm this hypothesis, overall, these results indicate that measuring cytokines might help identify children at high risk of suffering RSV complications; this would allow starting treatments in earlier stages of the disease. Also, among the genes that are over-represented during RSV infection are the STAT family members, which act as transcription activators in response to cytokines. For instance, the absence of *STAT1* resulted in airway dysfunction in knockout mice infected with RSV [32], and variation at *STAT2* has been associated with RSV susceptibility in preterm children [33].

Matrix metallopeptidases (MMPs) are proteins mostly known for degrading extracellular matrix proteins, but these are also known to be involved in other processes such as cytokine inactivation [34]. Experiments in mice carried out by Kong et al. [35] indicated that RSV infection raises MMP-9 levels, whereas the reduction of MMP-9 resulted in decreased viral replication, suggesting that MMP-9 may be a potential therapeutic target for RSV disease. Our results are in line with these findings as we found this gene up-regulated, supporting the hypothesis that MMP-9 could be a promising drug target.

Like other authors, we found up-regulated interferon-inducible genes such as *EIF2AK2* [5], which according to previous results plays a role in RSV susceptibility and immunological response. It has been described that mRNA level of this gene (and expression of *TLR4*) depends at least partially on the patient ethnicity, suggesting that the transcriptional response of individuals may be affected by the populational background [36,37,38]. Another interferon-induced gene we found up-regulated was *IFITM1*, which, according to Zhang et al. [39], inhibits RSV infection interfering with the viral entry and replication processes. Remarkably, our infected patients expressed as almost 50% more Interferon-induced GTP-binding protein Mx1 gene (log_2_ FC = 1.48) than the healthy controls. This finding is interesting as it has been argued that polymorphisms in *MX1* predispose to severe form of RSV in infants [36].

We also found an up-regulation of genes belonging to the innate immune response. For instance, *OAS1* and *OAS2* are responsible for the activation of ribonuclease L, which is part of the innate immune defense, during viral infection [40]. Ribonuclease L is an interferon IFN-induced ribonuclease that, when activated, destroys all single-stranded RNAs within the cell (cellular and viral) including rRNAs [41]. The destruction of all RNA within the cell is its last attempt to fight back against a virus before the onset of apoptosis [42].

RSV infection also induces an over-representation of DEG related to the neutrophil degranulation pathway; this pathway was found to be related to the immune response to RSV [43] and other viral respiratory diseases [44]. It has been shown that neutrophils degranulate into the airway in response to RSV, pointing to a local innate response to the infection. Neutrophils degranulation implies the release of antimicrobial substances that may contribute to the control of commensal bacteria in the upper respiratory tract [28].

According to our meta-analysis, five pattern recognition receptors (*CD14*, *TLR4*, *TLR5*, *TLR7* and *TLR8*) are overexpressed during RSV infection. Variants at these genes might be associated with the development of RSV bronchiolitis in different human populations, including Israel, Finland, Argentina, Japan, and Greece [45,46,47,48,49,50,51]. In 2000, Kurt-Jones et al. [24] reported that RSV persisted longer in the respiratory organs of infected *TLR4*-deficient mice in comparison to controls, and suggested that therapies that target the expression of TLRs could be useful to fight RSV infections [24]. Moreover, *TLR4* polymorphisms Asp299Gly and Thr399Ile seem to be associated with an enhanced risk of developing severe RSV bronchiolitis [45]. Furthermore, Zhou et al. [52] indicated that the *TLR4* signaling pathway, in conjunction with *MYD88* up-regulation, could be responsible for the activation of immune responses to RSV infection in airway epithelial cells [52]. Our results are in agreement with their observations, as we detected the up-regulation of both genes in the blood of RSV-infected patients when compared to healthy controls (Table S2).

The up-regulation of the *TLR3* cascade is particularly interesting since this receptor recognizes viral dsRNA, ultimately stimulating the production on type I interferons, a family of cytokines that regulate immune response to viral and other intracellular infections [53].

The over-representation in patients of C-type lectin receptors (CLRs) pathway (R-HSA-5621481; Table S3) is also remarkable as it has been described that the detection of viral glycans by these receptors help to fight viral infections. Up-regulation of CLRs activates antiviral immune responses such as antigen processing and presentation, T cell activation and phagocytosis, suppressing viral dissemination within the host. Nevertheless, CLRs can be a double-edged sword as some viruses have evolved to use these receptors for viral entry into host cells, avoiding immune recognition, where kidnapping the host cell machinery they produce hundreds of new copies of themselves spreading their copies further into the host [54].

Whole exome sequencing studies have revealed host biomarkers of susceptibility to RSV belonging to the olfactory and taste receptors. Single nucleotide polymorphism (SNP) variation at these genes has been observed to be associated with RSV infection [8]. RSV has also been described to cause post-viral olfactory loss [55]. According to our meta-analysis, RSV infection also has an impact on the DEG related to on the olfactory signaling pathway (R-HSA-381753; adjusted *p*-value = 1.09 × 10^−12^).

Last but not least, we identified a 17-transcript blood signature specific for RSV that differentiates it from other respiratory viruses of similar etiology such as influenza, rhinovirus, coronavirus and enterovirus. This indicates that RSV alters the host transcriptome in a specific manner that can be distinguished from other respiratory viruses. The identified signature could be relevant for RSV diagnosis. Even though current automatic PCR-based technologies already show good performance [56], these 17 transcripts might further our understanding of host molecular processes specifically altered by the RSV, which could eventually lead to the discovery of new drug targets for the treatment of RSV patients. Future laboratory validation will be necessary before the discovered signature can be used in clinical settings.

A major advantage of the present meta-analysis is that, by gathering data from different studies, we substantially increase sample size and consequently the statistical power to detect DEG when compared to controls and other pathogens and reduce the potential bias derived from selecting patients in particular populations, geographic areas and/or seasons. To reduce batch effects the data were carefully normalized and preprocessed (see Methods section); a limitation of this procedure is however that it not only reduces artificial sources of variability between datasets, but it might also reduce biological sources. The normalization procedure also reduces the number of probes analyzed to those that are shared between all microarrays. Another limitation of our study is that information on the RSV serotype (A/A2 or B), neither the disease status of the patients, are not available for all the datasets.

The present study is a stepping-stone towards understanding how RSV affects the host transcriptome, but further studies are needed to better understand how the host transcriptomic response change among serotypes, and during the progression of the disease. These studies would allow to shed further light in the mechanism responsible of RSV pathogenicity.

## 4. Methods

### 4.1. RSV Transcriptomic Datasets

We queried the public gene expression microarray repository Gene Expression Omnibus (GEO) for human gene expression datasets using the following terms: “RSV” and/or “syncytial”. We retained only those studies containing microarray expression data from whole blood samples of RSV infected patients. Eight databases were identified:

GSE42026 [52]: including 33 healthy controls, 19 influenza, and 22 RSV.

GSE34205 [57]: including 22 healthy controls, 28 influenza, and 51 RSV.

GSE106475 [58]: including 18 healthy controls, 21 influenza, 4 rhinovirus, and 4 RSV.

GSE77087 [54]: including 23 healthy controls, and 81 RSV.

GSE80179 [54]: including 52 healthy controls, and 27 RSV.

GSE117827 [59]: including 6 healthy controls, and 4 RSV.

GSE38900 [14]: including 39 healthy controls, and 130 RSV.

GSE68310 [60]: including 4 coronavirus, 4 enterovirus, 125 healthy controls, 120 influenza, 5 influenza plus coronavirus, 68 influenza plus rhinovirus, 67 rhinovirus, 16 rhinovirus plus coronavirus, 4 rhinovirus plus enterovirus, and 4 RSV.

More information on the datasets used is provided in Table S1.

### 4.2. Datasets Merging, Raw Data Normalization and Assessment of Differentially Expressed Transcripts

To merge and integrate the public domain RSV microarray studies, we first normalized and preprocessed each dataset separately using the package Lumi [61] for Illumina^®^ microarrays data and the package Oligo [62] for Affymetrix^®^ datasets (Figure S2). We also conducted a principal component analysis (PCA) to check for outliers and evaluate the presence of strong batch effects that could affect the analysis of the data.

Subsequently, we used the R package COCONUT (COmbat CO-Normalization Using conTrols) to combine all datasets into one and reduce batch effects in the meta-analysis [21].

**Figure 4:**
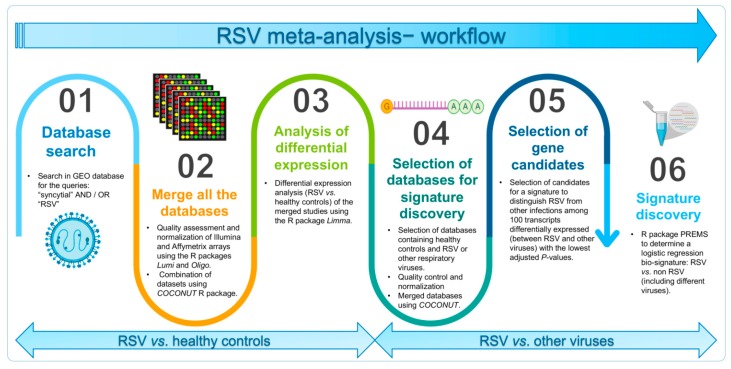
Scheme of the methodological procedure.

Finally, to determine which genes are significantly up- or down-regulated during the RSV infection (*n* = 296) when compared to both healthy controls (*n* = 193) and other viral conditions as a single group (*n* = 360; Table S2), we used the R package limma [63] and a moderated t-statistic. A linear model was fitted considering the age as a categorical covariate of the model (children / adult); this allowed us to minimize confounding effects considering that the study GSE68310 was carried out on adults whereas the rest of studies were conducted in children. Multiple testing correction was performed using the False Discovery Rate (FDR). A summary of the methodological procedure is shown in Figure 4.

### 4.3. Pathway Analysis

We used the Reactome pathway database to examine biological pathways associated with the genes differentially expressed in RSV patients. Thus, to categorize differentially expressed genes (DEG) for overrepresentation of Reactome pathways we used PANTHER Classification System with the following parameters: Reactome pathways overrepresentation test (released on 2019-03-08) with Reactome version 65 (released on 2019-03-12), and *Homo sapiens* as a reference list. Statistical significance was evaluated using Fisher’s exact test and FDR as the multiple test correction method.

### 4.4. Signature Discovery Using Parallel Regularized Regression

Our hypothesis is that RSV affects the transcriptome in a particular way distinguishable from other viral conditions. From the list of DEG obtained when compared RSV patients against other viral conditions (Table S2), we investigated a minimum specific transcript signature of RSV infection. We used Parallel Regularised Regression Model Search (PReMS) [64] in a randomly split dataset removing the healthy controls: training set (*n* = 521) and validation set (*n* = 135). PReMS explores different logistic regression models constructed from optimal subsets of the candidate genes while increasing the model size iteratively. PReMS [64] was chosen instead of other methods because it tends to select signatures with fewer genes without sacrificing model accuracy, which would facilitate its future translation into a clinical test [17]. The bio-signature was searched among the top 100 DEG between RSV and non-RSV categories.

Finally, the accuracy of the model estimated by PReMS was calculated as the area under the receiver operator curve (AUC) using the R package pROC [65] in both training and test cohorts.

All analyses were carried out using R software version 3.5.2 [66].

## 5. Conclusions

The present integrated multicohort analysis suggests that RSV alters the expression of >1500 genes in the host. A number of them are related to different pathways, namely cell cycle, immunological response to viral infection (including pattern recognition receptors), and olfactory signaling. In addition, RSV modifies the host transcriptome in a very specific manner, different from other respiratory viruses with similar phenotypes. We found a 17-transcript signature (validated in several independent cohorts), that allows the discrimination of RSV infection from other respiratory viruses. Considering the small number of transcripts involved, this signature might be potentially translated into a point of care test.

This study is a step forward to a better understanding of the molecular mechanism underlying RSV infection. The biomarkers of RSV infection detected may help discover new drug targets and improve the development of vaccines. Using pathway-based approaches such as GO term enrichment or Ingenuity pathway analysis (IPA) to prioritize the genes whose expression is altered by RSV infection may help discovering new drug targets and improve the development of vaccines.

## Figures and Tables

**Table 1:** Genes Included in the respiratory syncytial virus (RSV) signature. LRC = logistic regression coefficient.

Gene Symbol	Gene Name	LRC
*MTX1*	Metaxin 1	0.8982
*HERC4*	HECT And RLD Domain Containing E3 Ubiquitin Protein Ligase 4	−0.4769
*NREP*	Neuronal Regeneration Related Protein	−1.0717
*MTM1*	Myotubularin 1	−0.9309
*CA4*	Carbonic Anhydrase 4	−0.2805
*PGLYRP1*	Peptidoglycan Recognition Protein 1	−0.2079
*ATP6V0B*	ATPase H+ Transporting V0 Subunit B	0.8723
*TMEM123*	Transmembrane Protein 123	0.4303
*MAP3K8*	Mitogen-Activated Protein Kinase Kinase Kinase 8	−0.9468
*OAS3*	2′-5′-Oligoadenylate Synthetase 3	−0.9468
*TMEM184B*	Transmembrane Protein 184B	0.8642
*HIST1H1C*	Histone Cluster 1 H1 Family Member C	0.4563
*RPAP3*	RNA Polymerase II Associated Protein 3	0.9074
*MMP9*	Matrix Metallopeptidase 9	0.2811
*GABARAPL1*	GABA Type A Receptor Associated Protein Like 1	0.6837
*RAB8B*	Ras-Related Protein Rab-8B	−0.7833
*TCL1B*	T Cell Leukemia/Lymphoma 1B	0.6148

**Table 2:** AUC values for different pathogens (in round brackets the 95% CI for 2,000 bootstrap replicates). TR = training cohort; TE = test cohort; YT = Youden threshold. NA = not available (due to limited number of samples)

Comparison	Cohort	AUC (95%CI)	Sensitivity	Specificity	YT	*n*
All pathogens vs. RSV	TR	0.9021 (0.8748–0.9293)	0.8129	0.9295	7.5455	521
Influenza vs. RSV	TR	0.8148 (0.7685–0.8612)	0.9336	0.6306	7.0509	383
Enterovirus vs. RSV	TR	0.9794 (0.9606–0.9981)	0.9735	1.0000	6.1804	229
Coronavirus vs. RSV	TR	0.7699 (0.4404–1.0000)	0.8983	0.6667	7.2816	229
Influenza + coronavirus vs. RSV	TR	0.9049 (0.8455–0.9643)	0.8673	1.0000	7.5455	230
Influenza + rhinovirus vs. RSV	TR	0.9643 (0.9353–0.9932)	0.9336	0.9231	7.0327	239
Rhinovirus vs. RSV	TR	0.9197 (0.8700–0.9693)	0.8761	0.9298	7.4474	283
Rhinovirus + coronavirus vs. RSV	TR	0.966 (0.9356–0.9963)	0.9602	0.9231	6.6594	239
Rhinovirus + enterovirus vs. RSV	TR	0.9631 (0.9244–1.0000)	0.9381	1.0000	6.8868	229
All pathogens vs. RSV	TE	0.8364 (0.7598–0.9131)	0.7356	0.8750	7.5925	135
Influenza vs. RSV	TE	0.7710 (0.6566–0.8853)	0.9429	0.5807	6.7334	101
Enterovirus vs. RSV	TE	0.9714 (NA-NA)	0.9714	1.0000	5.5263	71
Coronavirus vs. RSV	TE	0.9429 (NA-NA)	0.9429	1.0000	6.2578	71
Influenza + coronavirus vs. RSV	TE	0.9714 (NA-NA)	0.9714	1.0000	5.6360	71
Influenza + rhinovirus vs. RSV	TE	0.9571 (0.9073–1)	0.8714	1.0000	7.2056	76
Rhinovirus vs. RSV	TE	0.8122 (0.6726–0.9519)	0.7714	0.8570	7.7252	84
Rhinovirus + coronavirus vs. RSV	TE	0.8952 (0.7849–1.0000)	0.8000	1.0000	7.5925	73
Rhinovirus + enterovirus vs. RSV	TE	0.9429 (NA-NA)	0.9429	1.0000	6.7107	71

## Supplementary Materials

Supplementary materials can be found at https://www.mdpi.com/1422-0067/21/5/1831/s1. Table S1. Description of samples and cohorts included in the present study. Table S2. Genes differentially expressed according to R package limma. Table S3. Reactome pathway enrichment analysis results. Figure S1. PCA plot of the eight datasets initially selected for the meta-analysis. The data points clustering on the right side of the plot (PC1) correspond to the outlier transcriptome profiles of the RSV samples from the dataset GSE80179; the control samples in GSE80179 cluster with the other controls.
