# Supplementary figures and images for: A Meta-Analysis of Multiple Whole Blood Gene Expression Data Unveils a Diagnostic Host-Response Transcript Signature for Respiratory Syncytial Virus
Source: Int J Mol Sci. 2020 Mar 6;21(5):1831. doi: 10.3390/ijms21051831 (PMC7084441; doi:10.3390/ijms21051831)

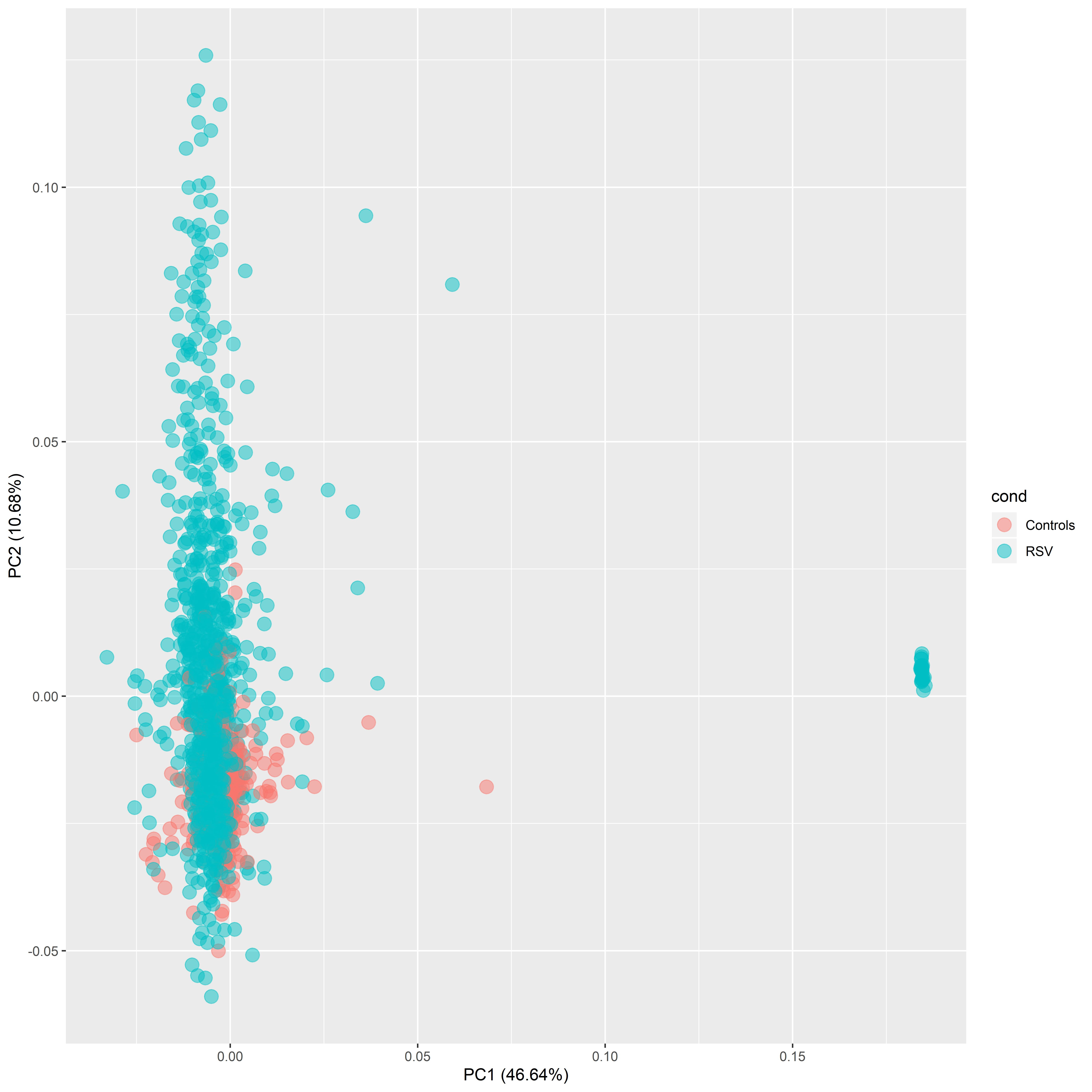

Supplement: Supplementary file 1 [file ijms-21-01831-s001.zip › ijms-727577-SI-for proofreading/Figure S1.tiff]

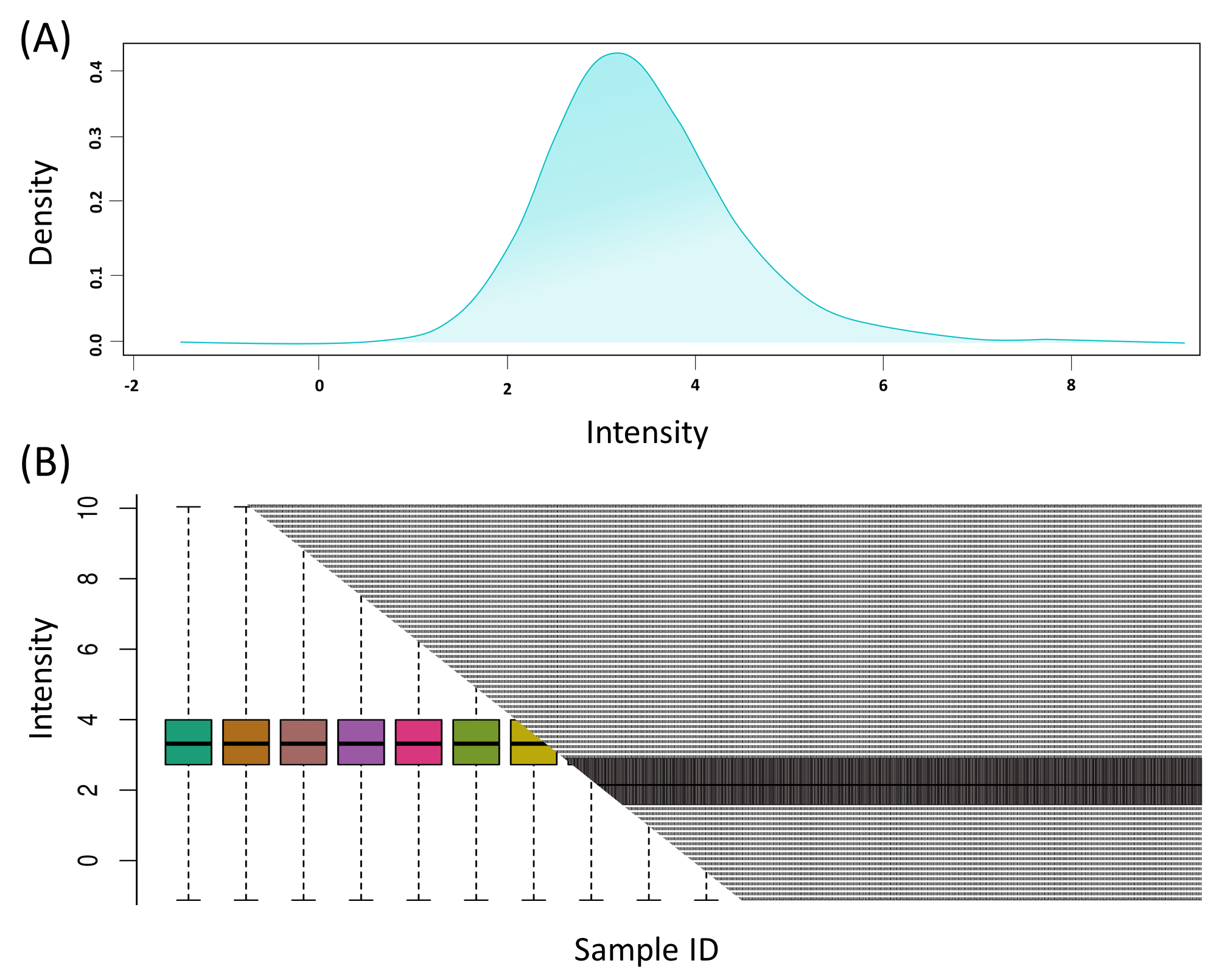

Supplement: Supplementary file 1 [file ijms-21-01831-s001.zip › ijms-727577-SI-for proofreading/Figure S2.tif]
